# Assessing the role of the T-box transcription factor Eomes in B cell differentiation during either Th1 or Th2 cell-biased responses

**DOI:** 10.1371/journal.pone.0208343

**Published:** 2018-12-06

**Authors:** Lucy Cooper, Lauren Hailes, Amania Sheikh, Colby Zaph, Gabrielle T. Belz, Joanna R. Groom, Kim L. Good-Jacobson

**Affiliations:** 1 Department of Biochemistry and Molecular Biology, Monash University, Clayton, Victoria, Australia; 2 Infection and Immunity Program, Biomedicine Discovery Institute, Monash University, Clayton, Victoria, Australia; 3 Walter and Eliza Hall Institute of Medical Research, Parkville, Victoria, Australia; 4 Department of Medical Biology, University of Melbourne, Parkville, Victoria, Australia; State University of New York Upstate Medical University, UNITED STATES

## Abstract

Successful T-dependent humoral responses require the production of antibody-secreting plasmablasts, as well as the formation of germinal centers which eventually form high-affinity B cell memory. The ability of B cells to differentiate into germinal center and plasma cells, as well as the ability to tailor responses to different pathogens, is driven by transcription factors. In T cells, the T-box transcription factors T-bet and Eomesodermin (Eomes) regulate effector and memory T cell differentiation, respectively. While T-bet has a critical role in regulating anti-viral B cell responses, a role for Eomes in B cells has yet to be described. We therefore investigated whether Eomes was required for B cell differentiation during either Th1 or Th2 cell-biased immune responses. Here, we demonstrate that deletion of Eomes specifically in B cells did not affect B cell differentiation in response to vaccination, as well as following viral or helminth infection. In contrast to its established role in CD8^+^ T cells, Eomes did not influence memory B cell differentiation. Finally, the use of an Eomes reporter mouse confirmed the lack of Eomes expression during immune responses. Thus, germinal center and plasma cell differentiation and the formation of isotype-switched memory B cells in response to infection are independent of Eomes expression.

## Introduction

Molecular regulation of B cell differentiation is critical for effective formation of humoral immunity to an infecting pathogen. Humoral immunity is underpinned by memory B cells and long-lived plasma cells [1]. During a T-dependent humoral immune response, B cells that recognise antigen can differentiate into early plasmablasts, or form germinal centers. Within germinal centers, they undergo rounds of somatic hypermutation and proliferation to produce high-affinity clones that are selected to exit the germinal centers and differentiate into memory B cells and plasma cells; the latter which migrates to, and resides within, the bone marrow to provide long-term immunity [1, 2]. Transcription factors are critical regulators of immune cell differentiation during an immune response. Within the B cell lineage, the transcription factors Bcl-6 and Blimp-1 are important for differentiation of B cells into germinal centers and plasma cells, respectively [3–6]. In contrast, there is no known transcription factor unique to memory B cell differentiation.

Transcriptional regulators are also integral in the tailoring of immune responses to different types of infection. Both B and T helper (Th) cells respond to signals in the pathogen-induced microenvironment that promote an effector response specialized to the infecting agent [7, 8]. Cytokines secreted by polarized Th cells in turn direct B cell behaviour by activating the expression of transcription factors that can mediate immunoglobulin isotype switching and other specialized transcriptional programs [8–10]. For example, B cells upregulate T-bet, switch to IgG2a/c [11] and express the chemokine receptor CXCR3 and induce other T-bet-dependent transcriptional changes [9] in response to IFNγ; this is repressed by the transcription factor c-Myb [12]. The transcription factor NFIL3 regulates IL-4-dependent switch to IgE [13], whereas RORα regulates IgA memory B cells [14]. It is unknown whether there are other transcription factors that underpin specialization of B cell responses to different Th cell-biased responses. Understanding the role of individual transcription factors, the relationship between transcriptional networks, and the pathogen-induced signals that regulate these transcription factors, will be important in designing vaccines for infectious agents for which an effective vaccine is currently lacking.

The T-box transcription factors T-bet and Eomes play important roles in multiple different immune lineages [15, 16]. T-bet and Eomes are involved in the differentiation of natural killer cells [17, 18], Th1 cells [19] and type 1 regulatory T cells [20]. However, the most well studied roles and relationship between T-bet and Eomes is within CD8^+^ T cells [16, 21–24], and particularly the bifurcation of their roles in regulating fate decisions of CD8 T cells [23, 25]. While it is well-known that T-bet is critical for B cell responses to viral infection [9, 12], there is no known study to date investigating whether Eomes regulates B cell differentiation in response to either Th2 or Th1 cell-biased infections.

To investigate whether Eomes was required for B cell differentiation or the formation of humoral memory, we generated mice in which Eomes was specifically deleted in B cells. Furthermore, we employed a number of immunization and infection models to assess whether Eomes was involved in tailoring B cell responses to different types of Th cell-biased responses. In summary, we determined that, unlike multiple other immune cells, differentiation of B cells into germinal center, plasma cell and isotype-switched memory B cells is independent of Eomes in these models.

## Materials and methods

### Mice, immunizations and purification of cells

*Cd23*-Cre [26] were provided by Meinrad Busslinger, and *Eomes*^*fl/fl*^ [20] and *Eomes*^*mcherry/+*^ [27] mice were provided by Gabrielle Belz. Animal procedures were approved by Monash University Animal Ethics Committee and all mice were maintained at the Monash Animal Research Platform. Mice were humanely euthanased by hypercapnea. *NP-KLH*: mice were injected intraperitoneally with 100μg of NP conjugated to KLH (molar ratio between 13–20), precipitated on 10% alum. *Influenza infections*: Mice were inoculated intranasally with 25uL of 250pfu HKx31 (H3N2) influenza virus as previously described [28, 29]. *Anasthesia*: Isofluorane (2.5%) was used to lightly anasthetize mice for intranasal infections. Mice were monitored to ensure stabilization of breathing and were warmed by a lamp to alleviate suffering if necessary. *Trichuris muris infection*: mice were infected via oral gavage with 200 *Trichuris Muris* eggs [30, 31].

### Flow cytometry and antibodies

Single cells were resuspended in PBS 2% FCS and stained for flow cytometric analysis. The following antibodies were used for flow cytometry: CD95 (JO2), IgG1 (X56), CD138 (281–2), IgG2a (R2-40), IgD (11-26c.2a), CD8 (53–6.7), CD44 (IM&), FVS fixable viability stains from BD; B220 (RA36B2), IgD (11-26c.2a), CD38 (90) and CD138 (281–2) from Biolegend; CD4 (GK1.5–7) and NIP were conjugated in-house. FcγRII/III (24G2; supernatant) and normal rat serum (Sigma-Aldrich) was used to block non-specific binding. FVS fixable viability stain was diluted 1:1000, 50ul was added to each cell sample, followed by incubation for 20 minutes at room temperature in the dark. Samples were then washed with 1 ml PBS 2% FCS, centrifuged at 1500 rpm for 5 mins at 4°C and each sample was resuspended in 50 μl of the antibody stain and incubated at 4°C for 15 minutes. Samples were then washed, centrifuged and resuspended in 200ul PBS 2% FCS. Cells were analyzed on a BD Fortessa or LSRIIa and the data analysed with FlowJo (Tree Star). *Sort-purification*: cells were stained with antibodies and purified by FACS Influx (BD).

### ELISpot analysis

The frequency of NP^+^IgG1^+^ antibody-secreting cells were assessed by an ELISpot assay. Briefly, multiscreen HA plates (Millipore) were coated with NP_12_BSA (10 μg/mL) overnight at 4°C. The next day, plates were blocked with PBS 1%BSA for one hour, after which plates were washed with PBS and samples prepared in RPMI 5% FCS, 50μM 2-Mercaptoethanol and 2mM Glutamine were added and incubated overnight at 37°C. Plates were then washed with PBS and PBS/Tween followed by incubation with secondary antibody (IgG1) directly conjugated to alkaline phosphatase (Sigma-Aldrich) for one hour. Plates were washed and developed with BCIP (Southern Biotech).

### Statistical analysis

Statistical analyses were performed using GraphPad Prism software and the Mann-Whitney non-parametrical statistical test was used to determine significance.

## Results and discussion

### Eomes is not required for B cell differentiation post-immunization with a model antigen

During a T-dependent immune response, antigen-specific B cells will either undergo differentiation into extrafollicular plasmablasts or generate germinal centers. To determine whether Eomes plays a role in B cell activation, differentiation into plasmablasts or the formation of germinal centers during a T-dependent immune response, we utilized the model antigen NP-KLH precipitated in alum. This model has been extensively used over the last few decades to determine the roles of transcription factors in B cell development or differentiation. To directly test the role of Eomes in B cells, we generated mice in which Eomes was conditionally deleted within mature B cells. Mice with exons of 2 and 3 of the gene encoding *Eomes* flanked by *loxp* sites were crossed with mice in which Cre recombinase is under control of the mature B cell-specific *Cd23* promoter (*Eomes*^*f/f*^*Cd23*^cre/+^ mice).

**Fig 1:**
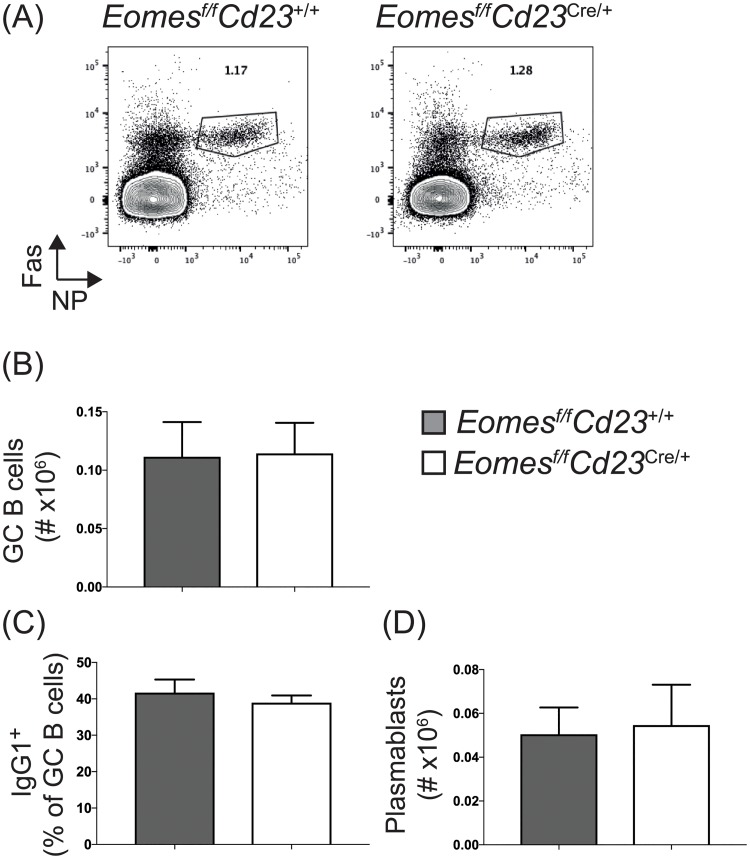
Eomes is not required for B cell differentiation in response to immunization with NP-KLH in alum. *Eomes*^*f/f*^*Cd23*^cre/+^ and littermate controls were immunized with NP-KLH precipitated in alum. Splenic B cell subsets were assessed at day 14 post-immunization. (A) Representative plots of antigen-specific germinal center B cells (NP^+^Fas^+^ B cells). Germinal center B cell number (B), the frequency of germinal center B cells that have switched to IgG1 (C) and plasmablast number (D) are shown. Data are pooled from two experiments, n = 5 mice per genotype, and is representative of a third independent experiment. Error bars indicate mean ± SEM. No significant difference was detected using the Mann-Whitney non-parametric test.

*Eomes*^*f/f*^*Cd23*^cre/+^ mice or littermate controls were immunized with the Th2 cell-biased NP-KLH in alum. Splenic B cell responses were assessed at 14 days post-immunization (Fig 1). Antigen-specific germinal center B cells (NP^+^Fas^+^ B cells) were at an equivalent frequency (Fig 1A) and number (Fig 1B) between *Eomes*^*f/f*^*Cd23*^cre/+^ and control mice. Furthermore, the frequency of germinal center B cells that had switched to IgG1, the dominant isotype for this response, was unaffected in the absence of Eomes (Fig 1C). Lastly, splenic plasmablasts were also at comparable numbers to control mice (Fig 1D). Together, these results suggest that Eomes is not required for B cell activation, proliferation and differentiation following immunization with NP-KLH in alum *in vivo*.

### Anti-influenza B cell responses do not require Eomes

**Fig 2:**
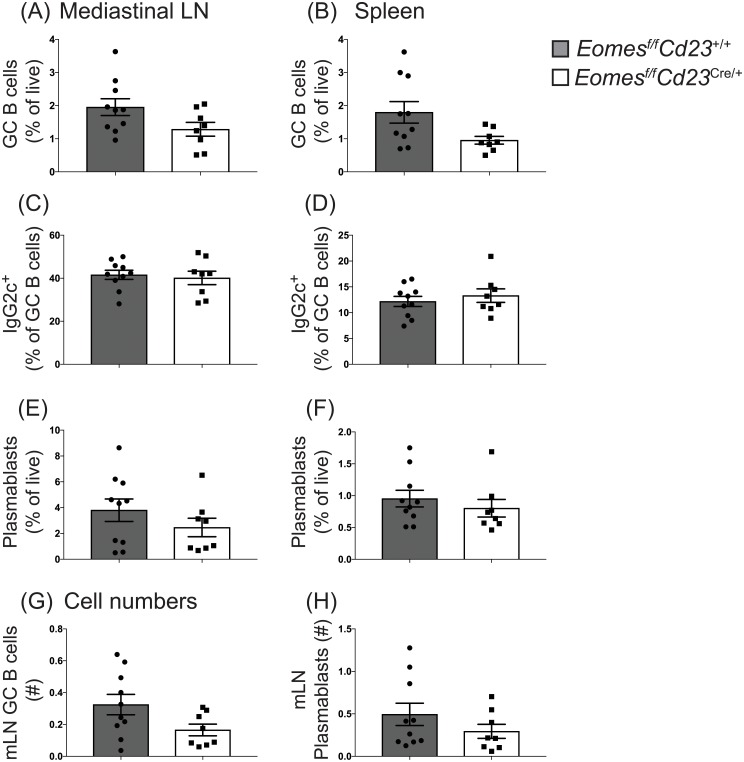
Eomes is not required for germinal center B cell formation or IgG2c isotype switching during influenza infection. *Eomes*^*f/f*^*Cd23*^cre/+^ and littermate controls were infected with HKx31 influenza virus and mediastinal lymph node-derived and splenic B cells were analyzed 8 days post-infection. (A-B) Mature activated B cells (B220^+^ IgD^lo^) were stained to identify germinal center B cells (CD95^hi^ CD38^lo^). (C-D) The frequency of germinal center B cells that had switched to IgG2c was assessed. (E-F) Frequency of the plasmablast population in either mediastinal lymph nodes (E) or spleen (F). (G-H) Total numbers of germinal center B cells (G) and plasmablasts (H) in the mediastinal lymph node. Data are pooled from 3 experiments, n = 8–10 per genotype. Error bars indicate mean ± SEM. No significant difference was detected using the Mann-Whitney non-parametric test.

Both T-bet and Eomes are critical for promoting CD8 T cell differentiation in response to viral infection. While T-bet plays a clear role in B cell differentiation during immune responses to viral infection [9, 12], a role for Eomes has not yet been tested. We therefore investigated whether Eomes was required for B cell differentiation in response to infection with influenza A virus. *Eomes*^*f/f*^*Cd23*^cre/+^ mice or littermate controls were infected with HKx31 (H3N2) influenza A virus and acute responses assessed at day 8 post-infection (Fig 2). In the absence of Eomes, germinal center B cells (CD95^hi^CD38^lo^ B cells; Fig 2A and 2B) were able to form in both the draining lymph node and in the spleen. While there was a trend decrease in the frequency of germinal center B cells, this was not statistically significant. Similar to IgG1 in NP-KLH-immunized mice, IgG2c switching was also unaffected (Fig 2C and 2D). While germinal center formation occurred in the absence of Eomes, it was possible that the plasmablast population required Eomes for differentiation. However, flow cytometric analysis demonstrated that plasmablast differentiation was also unaffected in the absence of Eomes (Fig 2E and 2F). Finally, no significant difference was detected in the total numbers of germinal center B cells and plasmblasts (Fig 2G and 2H). Therefore, Eomes was not required for the formation of B cell responses to a Th1 cell-biased viral infection.

### B cell differentiation during Th2 cell-dependent immunity to *Trichuris muris* is independent of Eomes

**Fig 3:**
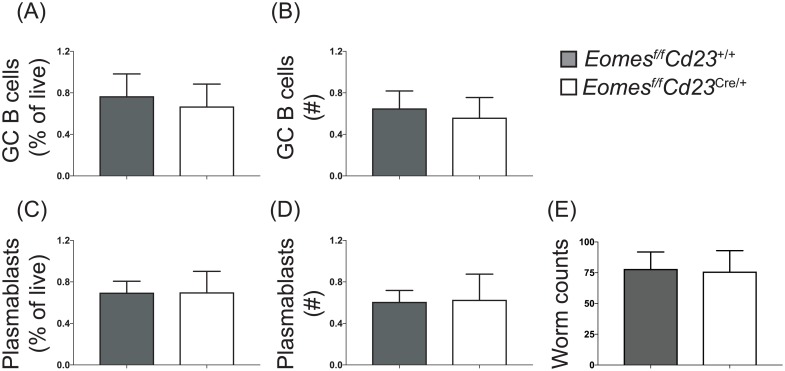
B cell differentiation in response to the Th2 cell-biased infection with *T*. *muris* does not require B cell-intrinsic Eomes expression. *Eomes*^*f/f*^*Cd23*^cre/+^ and littermate controls were infected with *T*. *muris*. At 21 days post-infection, B cell responses in the mediastinal lymph nodes (A-D) and the number of worms present in the colon (E) were assessed. Germinal center B cell frequency (A) and number (B), as well as plasma cell frequency (C) and number (D), are shown. (E) The colon from each mouse was frozen and the number of worms still present counted. Data is representative of 3 experiments performed 3–4 weeks post-infection, n = 5 per genotype. Error bars indicate mean ± SEM. No significant difference was detected using the Mann-Whitney non-parametric test.

Our data thus far demonstrates that B cell differentiation proceeds in the absence of Eomes in immunized mice or those infected with influenza. To complement these studies, a Th2-cell biased parasitic infection model was utilized. *Eomes*^*f/f*^*Cd23*^cre/+^ mice or littermate controls were infected with the helminth *Trichuris muris*. B cell responses were assessed at day 21 post-infection, a time point at which wild-type mice have generated germinal center B cell responses (Fig 3). Both the frequency and number of germinal center (Fig 3A and 3B) and plasmablast (Fig 3C and 3D) populations were comparable in Eomes-deficient and Eomes-sufficient mice. Lastly, worms were cleared to an equivalent extent in both *Eomes*^*f/f*^*Cd23*^cre/+^ mice and littermate controls. Together, these data demonstrate that B cell-intrinsic expression of Eomes was not required for B cell differentiation during helminth infection.

### Memory B cell formation is independent of Eomes in either Th1 or Th2 cell-biased responses

**Fig 4:**
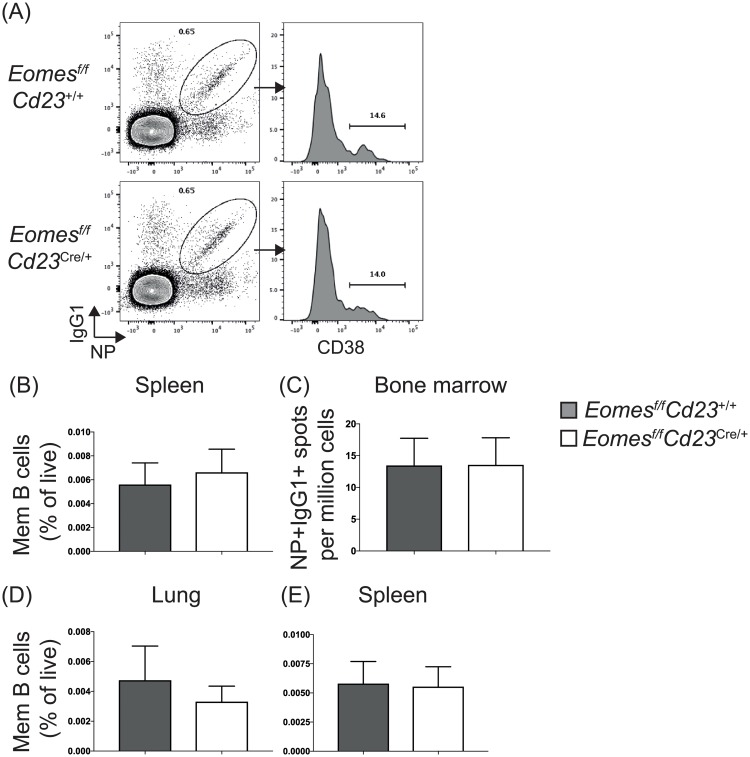
Memory B cell formation in either Th1 or Th2 cell-biased responses proceeds in the absence of Eomes in B cells. *Eomes*^*f/f*^*Cd23*^cre/+^ and littermate controls were immunized with NP-KLH precipitated in alum (A-C) or infected with influenza (D-E) to assess B cell memory formation. (A) Representative plots of antigen-specific memory B cells (NP^+^IgG1^+^CD38^+^ B cells) in the spleen; representative of 5 mice at d14 and 5 mice at d28 post-immunization. (B) Summary plot of antigen-specific memory B cell frequency. (C) NP-binding IgG1-secreting B cells in the bone marrow was analyzed via ELISpot at d28 post-immunization; data is from 2 experiments, n = 5 per genotype. Error bars indicate mean ± SEM. No significance was detected using the Mann-Whitney non-parametric test. (D-E) HA^+^CD38^+^IgG2c^+^ B cells in the lung (D) or spleen (E) were assessed in influenza-infected mice 28 days post-infection. Data is from 2 experiments, n = 6–8 per genotype for splenic samples and n = 4–5 per genotype for lung samples. Error bars indicate mean ± SEM. No significance was detected using the Mann-Whitney non-parametric test.

Given the role of Eomes in CD8 T cell memory formation, we investigated the formation of Eomes in B cell memory formation. *Eomes*^*f/f*^*Cd23*^cre/+^ mice or littermate controls were either immunized with NP-KLH in alum (Fig 4A and 4C) or infected with influenza (Fig 4D and 4E). Antigen-specific memory B cell formation was assessed 4 weeks post-immunization or post-infection. In NP-KLH-immunized mice, the frequency of memory B cells (defined as NP^+^IgG1^+^CD38^+^ B cells) was equivalent between Eomes-sufficient and Eomes-deficient mice (Fig 4A and 4B), as were the number of bone marrow-resident, long-lived plasma cells (Fig 4C). Similarly, in mice infected with influenza, the formation of memory B cells (defined as HA^+^CD38^+^IgG2c^+^) was also unaffected (Fig 4D and 4E). Therefore, unlike CD8 T cells, memory B cell formation is independent of Eomes.

### Eomes expression is not detected in B cells post-infection

**Fig 5:**
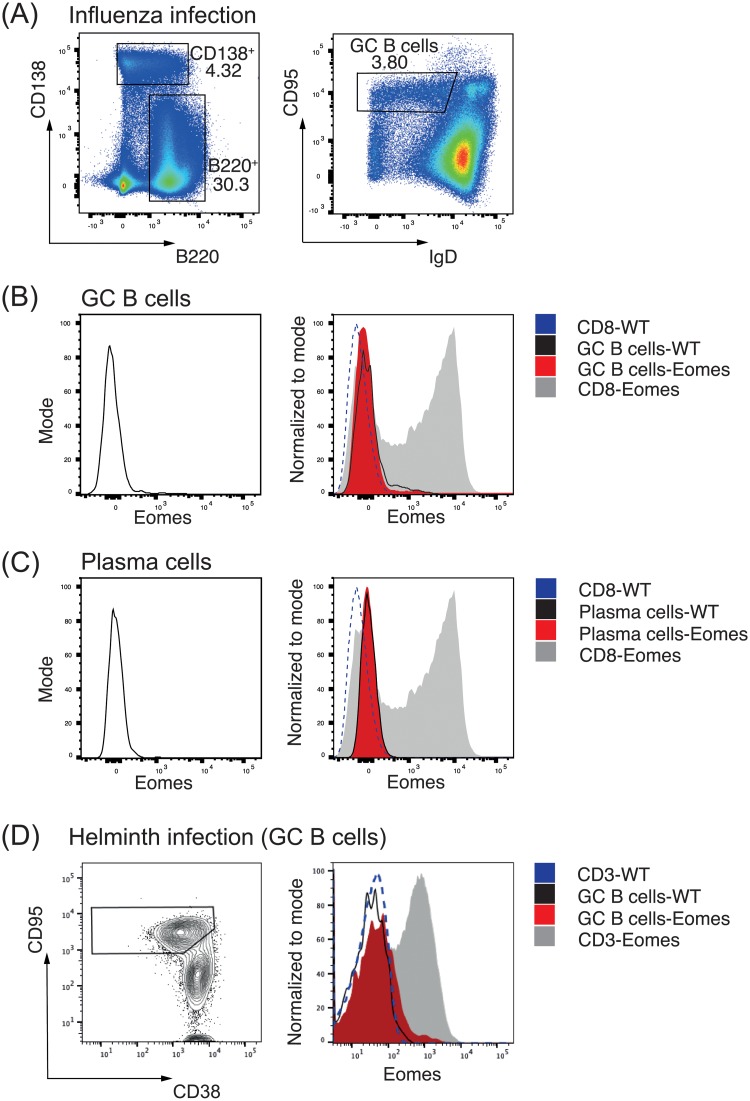
Eomes is not detected in germinal center B cells during either Th1 or Th2 infection. (A-C) Representative flow cytometric plots of influenza-infected *Eomes*^*mcherry/+*^ (reporting for *Eomes* mRNA transcript) or C57BL/6 mice. Splenic germinal center B cells (A, B), plasma cells (A, C) and CD8 T cells (B-C) were assessed for *Eomes* expression 8 days post-infection. CD8 T cells from C57Bl/6 mice are represented by the dotted line. (D) Representative flow cytometric plots of mice that were infected with *T*. *muris* and assessed for *Eomes* expression in germinal center B cells and CD3^+^ T cells 21 days post-infection. *Eomes*^*mcherry/+*^ data are representative of ≥3 mice per experiment.

Finally, we examined the expression of Eomes within B cells to confirm that Eomes does not play a role in B cells during responses to models of either Th1 or Th2 cell-biased infection. To do this, we utilized *Eomes*^*mCherry/+*^ mice, in which *Eomes* mRNA transcript is reported by mCherry fluorescence [20]. Mice were infected with either influenza (Fig 5A–5C; assessed 8 days post-infection) or *T*. *muris* (Fig 5D; assessed 21 days post-infection) and mCherry fluorescence was examined in splenic germinal center B cells, plasma cells and CD8 T cells. Eomes was not detected in germinal center B cells or plasma cell populations, while activated CD8 T cells were positive for Eomes expression [16]. Thus, these results are consistent with our findings that fail to identify a role for Eomes in germinal center or plasma cell differentiation.

Transcription factors are critical regulators of immune cell differentiation during responses to immunization or infection. A number of transcription factors are utilized by different immune cell subsets for specialized functions. In particular, the T-box transcription factors T-bet and Eomes have important, interconnected roles in NK, Th and CD8 T cell biology. While T-bet is important for effective B cell responses to viruses such as influenza, it was unknown whether Eomes also contributed to an effective B cell response and/or the formation of humoral memory. By utilizing mice that have been conditionally deleted for Eomes in B cells, our data demonstrated that the deletion of Eomes had no significant effect on germinal center formation or the formation of humoral memory across multiple immunization and infection models.

Why do B cells utilize T-bet but not Eomes? One possibility is the differential modulation of H3K4me3 and H3K27me3, histone marks that are important for regulating both B and T cell differentiation [32, 33]. In GC B cells, the promoter for *Eomes* is solely marked by H3K27me3, a histone mark that condenses chromatin to repress gene expression. In contrast, *Tbx21* (the gene encoding T-bet) is also marked by H3K4me3, a permissive mark that poises genes for expression [32]. What factors may regulate this specific targeting are unclear. The link between changes in the microenvironment and modulation of histone marks is a critical, nascent area of research that may eventually shed light on context-specific gene regulation. In summary, while T-bet plays a similar role in B and T cells in driving tailored responses to viral infection, we have shown that Eomes does not play a role in the formation of germinal centers, plasma cells or isotype-switched memory B cells during responses to influenza, helminth or immunisation in mice.
